# Mental Health in Frontline Medical Workers during the 2019 Novel Coronavirus Disease Epidemic in China: A Comparison with the General Population

**DOI:** 10.3390/ijerph17186550

**Published:** 2020-09-09

**Authors:** Yiming Liang, Kankan Wu, Yongjie Zhou, Xin Huang, Yueyue Zhou, Zhengkui Liu

**Affiliations:** 1 CAS Key Laboratory of Mental Health, Institute of Psychology, Chinese Academy of Sciences, Beijing 100101, China; liangym@psych.ac.cn (Y.L.); wukk@psych.ac.cn (K.W.); huangx@psych.ac.cn (X.H.); zhouyueyue@psych.ac.cn (Y.Z.); 2 Department of Psychology, University of Chinese Academy of Sciences, Beijing 100049, China; 3 Research Center for Psychological and Health Sciences, China University of Geosciences, Wuhan 430074, China; qingzhu1108@126.com

**Keywords:** coronavirus, COVID-19, mental health, frontline medical workers, general public

## Abstract

Background: Since December 2019, China has been affected by a severe outbreak of coronavirus disease 2019 (COVID-19). Frontline medical workers experienced difficulty due to the high risk of being infected and long and distressing work shifts. The current study aims to evaluate psychological symptoms in frontline medical workers during the COVID-19 epidemic in China and to perform a comparison with the general population. Methods: An online survey was conducted from 14 February 2020 to 29 March 2020. A total of 899 frontline medical workers and 1104 respondents in the general population participated. Depression, anxiety, insomnia, and resilience were assessed via the Patient Health Questionnaire (PHQ-9), Generalized Anxiety Disorder Scale (GAD-7), Insomnia Severity Index (ISI), and abbreviated Connor–Davidson Resilience Scale (CD-RISC-10), respectively. Results: Overall, 30.43%, 20.29%, and 14.49% of frontline medical workers in Hubei Province and 23.13%, 13.14%, and 10.64% of frontline medical workers in other regions reported symptoms of depression, anxiety, and insomnia, respectively. In addition, 23.33%, 16.67%, and 6.67% of the general population in Hubei Province and 18.25%, 9.22%, and 7.17% of the general population in other regions reported symptoms of depression, anxiety, and insomnia, respectively. The resilience of frontline medical staff outside Hubei Province was higher than that of the general population outside Hubei Province. Conclusion: A large proportion of frontline medical workers and the general public experienced psychological symptoms during the COVID-19 outbreak. Psychological services for frontline medical workers and the general public are needed.

## 1. Introduction

Coronavirus disease 2019 (COVID-19) was first reported in Wuhan, China at the end of 2019. Hubei Province is the most severely affected area in China, and more than 68,000 COVID-19 cases have been reported there since December 2019. As of 8 March 2020, the Chinese government had sent 346 medical teams across the country in response to the COVID-19 outbreak, and a total of 42,600 medical workers have been sent to Wuhan city and Hubei Province to provide medical assistance since 24 January 2020 [1].

Some scholars noted that a major public health emergency could be regarded as an acute episode of a biological disaster and could thus result in a high rate of psychiatric morbidity [2]. Frontline medical staff are the first-line fighters against COVID-19. These workers were at extremely high risk of infection and under enormous work pressure for more than one month. They faced a protracted experience of multiple pressures, including isolation, high risk of infection, and long and distressing work shifts [3,4]. Previous research found that under high-risk and stressful situations during an epidemic, medical staff are prone to experience a range of psychological problems, including fear, depression, anxiety, post-traumatic stress symptoms, and insomnia [5,6]. For example, a large proportion of the medical staff who battled severe acute respiratory syndrome (SARS) suffered depression, anxiety, and sleep problems [2,7]. Therefore, the mental health status of the frontline medical staff fighting COVID-19 is particularly worthy of attention. 

A high proportion of the medical staff who are fighting COVID-19 around the world have suffered psychological problems during the COVID-19 epidemic [8,9,10]. Among 441 healthcare workers in Poland, 64.4%, 70.7%, and 58.0% presented symptoms of anxiety, depression, or insomnia, respectively [10]. Among 1422 Spanish health personnel, 58.6% and 46% had possible anxiety or depression, respectively, and 20.7% and 5.3% had severe anxiety or depression disorder [8]. China is the first country to be severely affected by COVID-19, and the mental health of Chinese frontline medical staff has been greatly affected. A considerable proportion of Chinese healthcare workers have reported symptoms of depression (25.2%–58%), anxiety (29.18%–54.2%), or insomnia (34.0%–36.1%) during the COVID-19 epidemic [11,12,13,14].

The COVID-19 pandemic has also led to an increased prevalence of symptoms of adverse psychological outcomes among the general public compared to that prior to the pandemic [9,15,16,17]. During the epidemic, the public was faced with mandatory quarantines, possible economic losses, and unexpected unemployment, all of which can increase anxiety, depression, and insomnia [10,16]. Previous studies reported that 14.6%–17.2% and 6.3%–28.8% of the Chinese general public displayed symptoms of depression or anxiety, respectively, during the epidemic [18,19,20]; these prevalences are lower than the prevalence of depression and anxiety among Chinese medical staff [11,13,14]. Compared to the general public, medical workers have been challenged by higher levels of exposure to the epidemic situation and by overwork [5,21]; thus, the mental health of medical workers may be more affected by COVID-19 than that of the general public. However, few studies have compared the mental health of medical workers with that of the general public. 

Previous studies showed that not all individuals exposed to stressful situations or even to traumatic events develop mental disorders [22,23]. Resilience, which is defined as the capacity to cope with and positively adapt to adversity, is an important protective factor and is of particular concern to researchers in the field of adversity [24,25]. Individuals with high resilience are better able to maximize internal resources (e.g., perseverance and self-efficacy) and external resources (e.g., social support) to mitigate the negative impact of adversity [26]. It has been demonstrated during the COVID-19 epidemic that resilience can help reduce worry, anxiety, and depression [8,27]. Moreover, medical staff have more knowledge about the epidemic and more professional work experience than the general public does; as a result, they may have more psychological endurance and confidence regarding COVID-19 [28]. Thus, the resilience of medical workers may be higher than that of the general public.

The aim of this study is to provide a preliminary psychological assessment of Chinese frontline medical staff involved in epidemic work and to compare their status with that of the general population to provide guidance for psychological assistance during the recovery period. We hypothesize that (i) the mental health of medical workers and of the general public has been greatly affected by the COVID-19 epidemic; (ii) the level of depression, anxiety, and insomnia symptoms is higher in medical workers than that in the general public; and (iii) the level of resilience is higher in medical workers than it is in the general public.

## 2. Method

### 2.1. Procedure and Participants

After the COVID-19 outbreak, on 28 January 2020, the comprehensive psychological action project known as the Ease Project was initiated by the Psychological Research Institute of the Chinese Academy of Sciences and the Chinese Psychological Society. The aim of this project was to provide psychological services to groups affected by COVID-19 and to reduce the psychosocial impact of the epidemic. To better understand the mental health states of the general population and of frontline medical workers, an online mental health survey was conducted. 

Due to the epidemic, face-to-face investigations were restricted. Thus, online questionnaires were administered via a WeChat applet. Data were collected both from the general population and from frontline medical workers during the COVID-19 pandemic from 14 February 2020 to 29 March 2020. The online questionnaire for medical workers was publicized through posters in hospitals and mobile cabin hospitals in Hubei and Hunan Provinces. We also asked frontline medical workers from other provinces who were working to support Wuhan to send the survey information to their home hospitals so that frontline medical workers from other provinces could participate in this survey. Ultimately, 899 participants from 28 provinces in mainland China completed the questionnaire. The regions with the most data sources were Hunan Province (233), Beijing City (168), and Hubei Province (138). Of the respondents, 224 were doctors, 406 were nurses, 69 were staff members in the medical technology department, 13 were anesthetists, 128 were assistant nurses, and 59 were administrative staff.

The online questionnaire for the general population was publicized and distributed through the Internet, and the adult participants volunteered to complete the survey. Ultimately, 1104 participants located in 31 provinces in mainland China completed the questionnaire. The regions with the most data sources were Shandong Province (405), Liaoning Province (199), Beijing City (186), and Hunan Province (112); 30 respondents were from Hubei Province. The questionnaires for both the general population and the frontline medical workers were administered via WeChat, and informed consent was also obtained from the participants via WeChat. After each respondent had given informed consent and his or her name had been recorded, a questionnaire was sent to the respondent. The study design and procedures were approved by the ethics review committee of the Institute of Psychology, Chinese Academy of Sciences on 21 February 2020 (Protocol number: A20021).

### 2.2. Measures

#### 2.2.1. Demographics

A questionnaire was devised to obtain the demographic information of the respondents in the general population and the frontline medical workers, including age, gender, marital status, education level, annual household income, and “if one or more relative or friend was infected by the virus”. For the frontline medical workers, additional information about their work situation was collected, including hours worked per day, years worked, and position.

#### 2.2.2. Patient Health Questionnaire (PHQ-9)

The Patient Health Questionnaire (PHQ-9) was used to assess symptoms of depression [29]. It is a 9-item self-report scale, and its items pertain to the Diagnostic and Statistical Manual of Mental Disorders criteria for major depressive disorder. The items are scored on a 4-point scale ranging from 0 to 3, and participants rate the items according their situation during the preceding two weeks. Previous literature suggests that total scores of 5–9 indicate mild depressive symptoms, and total scores ≥10 indicate moderate-to-severe depressive symptoms [29,30]. The reliability and validity of the simplified Chinese version of the PHQ-9 have been demonstrated [31]. In this study, the scale exhibited good internal consistency (Cronbach’s α of 0.91 in frontline medical workers and 0.89 in the general population).

#### 2.2.3. Generalized Anxiety Disorder Scale (GAD-7)

The Generalized Anxiety Disorder Scale (GAD-7) was used to assess anxiety symptoms and has been proven to be a valid and efficient tool for screening for GAD and assessing its severity [32]. It is a 7-item self-report scale, and the items are scored on a 4-point scale ranging from 0 to 3. Participants rate the items according their situation during the preceding two weeks. Total scores of 5–9 and ≥10 indicate mild and moderate-to-severe anxiety symptoms, respectively [33]. The reliability and validity of the simplified Chinese version of the GAD-7 have been demonstrated [34]. In this study, the scale exhibited good internal consistency (Cronbach’s α of 0.93 in frontline medical workers and 0.93 in the general population). 

#### 2.2.4. Insomnia Severity Index (ISI)

The Insomnia Severity Index (ISI) was used to assess insomnia and is composed of 7 items that evaluate (1) the severity of initial, middle, and late insomnia; (2) satisfaction with current sleep pattern; (3) sleep problem interference with daily functioning; (4) noticeability of impairment caused by the sleep problem; and (5) worry about sleep problems [35]. The items are scored on a 5-point scale ranging from 0 to 4. Participants rate the items according their situation during the preceding two weeks. Total scores of 8–14 and ≥15 indicate mild and moderate-to-severe insomnia, respectively [35]. In this study, the scale exhibited good internal consistency (Cronbach’s α of 0.94 in frontline medical workers and 0.92 in the general population).

#### 2.2.5. Abbreviated Version of the Connor–Davidson Resilience Scale (CD-RISC-10)

The abbreviated version of the Connor–Davidson Resilience Scale (CD-RISC-10) was used to assess resilience and is a useful instrument for this purpose [36]. The CD-RISC-10 was created from the CD-RISC [24] and translated into Chinese by Wang and his colleagues [37]. It is a 10-item self-report scale, and the items are scored on a 5-point scale ranging from 0 to 4. Higher total scores indicate a high level of resilience. Participants rate the items according their situation during the preceding month. In this study, the scale exhibited good internal consistency (Cronbach’s α of 0.96 in frontline medical workers and 0.96 in the general population).

### 2.3. Data Analysis

Participants were divided into 4 groups based on occupation (frontline medical workers and the general population) and province (Hubei Province and other regions). In the current study, descriptive statistics involved the mean value and standard deviation for continuous and nonnormally distributed data, and analysis of variance (ANOVA) was used to assess group differences. Bonferroni’s post hoc multiple comparison test was used to adjust *p*-values. Categorical variables were described using the frequency and percentage, and the chi-square test was used to assess group differences. Because the number of respondents from the general population in the Hubei Province group was much smaller than that in the other three groups, this group was excluded when conducting the group differences test. All statistical analyses were performed in SPSS (Version 22.0 for Windows).

## 3. Results

A total of 899 frontline medical workers (138 from Hubei Province; 761 from other regions) and 1104 respondents from the general population (30 from Hubei Province; 1074 from other regions) completed the survey. Table 1 presents the sociodemographic features of the frontline medical workers and the general population. Compared to the general population, frontline medical workers were more likely to be female, older, and married; to have a higher annual household income; and to have a higher education level. In addition, frontline medical workers were more likely to have one or more relatives or friends with COVID-19 (or who were quarantined).

The scores for depression, anxiety, insomnia, and resilience in the 4 groups are shown in Table 2, and the prevalence of depression, anxiety, insomnia, and their comorbidity in the 4 groups are shown in Table 3. For frontline medical workers in Hubei Province, medical workers in other regions, the general population in Hubei Province, and the general population in other regions, the prevalence of moderate/mild depression was 30.43/73.91%, 23.13/55.45%, 23.33/53.33%, and 18.25/46.09%, respectively; the prevalence of moderate/mild anxiety was 20.29/60.14%, 13.14/44.81%, 16.67/53.33%, and 9.22/32.22%, respectively; and the prevalence of moderate/mild insomnia was 14.49/43.48%, 10.64/29.70%, 6.67/23.33%, and 7.17/23.46%, respectively. Differences in the levels of depression, anxiety, insomnia, and resilience among the three groups of medical workers in Hubei Province (group 1), medical workers in other regions (group 2), and the general population in other regions (group 3) were compared by ANOVA. The results showed that the depression, anxiety, insomnia, and resilience levels of the three groups were significantly different (all *p* < 0.024). The results of multiple comparison tests showed significant differences between each group in depression (group 1 vs. group 2: *p* = 0.006; group 1 vs. group 3: *p* < 0.001; group 2 vs. group 3: *p* < 0.001), anxiety (group 1 vs. group 2: *p* = 0.004; group 1 vs. group 3: *p* < 0.001; group 2 vs. group 3: *p* < 0.001), and insomnia (group 1 vs. group 2: *p* = 0.008; group 1 vs. group 3: *p* < 0.001; group 2 vs. group 3: *p* = 0.001). The results of multiple comparison tests showed significant differences in resilience only between group 2 and group 3 (group 1 vs. group 2: *p* = 0.521; group 1 vs. group 3: *p* = 0.991; group 2 vs. group 3: *p* = 0.022). In general, the levels of depression, anxiety, and insomnia were highest among medical workers in Hubei Province, followed by medical workers in other regions, and they were lowest in the general population in other regions. The resilience of frontline medical workers in other regions was significantly higher than that of the general population in other regions.

## 4. Discussion

The current study enrolled 899 frontline medical workers (138 in Hubei province and 761 in other regions of China) and a comparison group of 1104 respondents from the general population (30 in Hubei province and 1074 in other regions of China) and revealed that both frontline medical workers in Hubei province and those in other regions reported a considerable prevalence of psychiatric symptoms. Medical staff in the Hubei area suffered more serious mental health problems than those in other regions. Overall, 30.43%, 20.29%, and 14.49% of frontline medical workers in Hubei Province reported symptoms of depression, anxiety, and insomnia, and 23.13%, 13.14%, and 10.64% of frontline medical workers in other regions reported symptoms of depression, anxiety, and insomnia, respectively.

A significant proportion of frontline medical workers in our survey experienced depression, anxiety, and insomnia symptoms. Several previous studies have also shown that medical staff experienced serious psychological problems during the COVID-19 outbreak [3,4,10,11]. The causes of the psychological problems experienced by frontline medical staff during the COVID-19 outbreak are complicated. A high risk of infection may make workers feel vulnerable or feel a loss of control over their health [2], especially because COVID-19 is highly infectious, has a high morbidity rate, and is potentially fatal [28,38]. Long hours of intense work, isolation from family, and a shortage of medical supplies also lead workers to feel distress [11].

The prevalence of depression, anxiety, and insomnia observed in our study is inconsistent with that in previous studies in which Chinese medical workers were sampled [11,39,40]. The main reasons for the differences may lie in the use of different measurement scales, reporting patterns, sampling times, and locations. With respect to reporting patterns, some studies reported any respondents with mild-to-severe symptoms [40], while others only reported respondents with moderate-to-severe symptoms [11]. After careful comparison, we found that the prevalence of depression, anxiety, and insomnia in our study is similar to that reported in Wang et al.’s study [41] but lower than that reported by Lai et al. [11] and Xiao et al. [39]. Lai et al. [11] found that 50.4%, 44.6%, and 34.0% of 1257 frontline health care workers reported symptoms of depression, anxiety, or insomnia, respectively, Xiao et al. [39] found that 54.2% and 58% of 958 healthcare workers had symptoms of anxiety or depression, respectively [39]. The differences in observed prevalence between our study and the cited studies may be due to differences in sampling time and place. Both Lai et al. [11] and Xiao et al. [39] collected data at the end of January during the peak of the Chinese epidemic, whereas our study and Wang et al.’s study [41] collected data from the end of February through March, a period during which the epidemic in China entered the plateau phase. Since March, the cumulative number of confirmed cases of COVID-19 in China has reached 80,000; cases from Hubei Province account for 84% of these, and the cumulative number of confirmed cases increased very slowly (approximately 100 cases per day nationwide) [41]. Previous studies have also shown that the prevalence of mental disorders decreases over time following traumatic events [42]. Thus, the alleviation of the COVID-19 epidemic and medical workers’ adaptation to the situation might have led to a reduction in the prevalence of depression, anxiety, and insomnia. Another possible reason for the difference in the results is the use of different sampling locations. Of the participants in Lai et al.’s [11] study and Xiao et al.’s [39] study, 60.5% and 73.6%, respectively, worked in Wuhan, and 20.8% and 14.6%, respectively, worked in Hubei Province outside Wuhan; these were the areas that were most severely affected by COVID-19. Our findings also indicated that during the COVID-19 outbreak, medical workers who worked in Hubei Province suffered more psychological symptoms than those who worked outside of Hubei Province. Wuhan and other areas in Hubei Province faced the most severe epidemic situation and adopted the strictest isolation measures [28,38]. The frontline medical staff there also faced the greatest work pressure and the greatest risk of infection. Therefore, the mental health problems of frontline medical staff in Wuhan and other areas of Hubei Province need the most attention.

Our research also found widespread symptoms of depression, anxiety, and insomnia in the general population, although the prevalence in this group was lower than that in the medical staff. Higher levels of exposure to the epidemic situation and overwork may be the main reasons for the poorer mental health of medical staff. It is worth noting that although the proportions of respondents who reported that a relative or friend had been infected with COVID-19 were low among both medical staff (3.34%) and the general public (0.72%), this event was still significantly higher among the medical staff than among the general public. Infection of family members or friends is a risk factor for mental health [15]. Having more relatives or friends who became infected may have led to poorer mental health among the medical staff than among the general population. Some previous studies also found that during the initial phase of the COVID-19 outbreak in China, a large proportion of the general population had psychiatric symptoms [9,16]. Their psychological response was due to multiple factors. First, the infectivity and prevalence of COVID-19 and the shortages of masks and health equipment made them worry about their health [28,38]. Second, the media’s coverage of the epidemic also exacerbated people’s fears [43]. Third, staying at home and social isolation greatly changed people’s lifestyles, and the closure of schools and business also led to negative emotions [43,44]. Therefore, the general population’s mental health is also worthy of attention, and psychological services need to be provided for them also.

Resilience has a protective effect that helps people withstand stressors and cope positively with adversity [45,46]. Our results indicated that the resilience of frontline medical staff outside Hubei Province was higher than that of the general population outside Hubei Province. Controllability is important in stress situations, and it can help individuals cope with stressful situations [47]. Medical staff have more knowledge about the epidemic and more professional work experience than the general population; thus, they may have more control and confidence regarding COVID-19 [28]. Furthermore, medical staff also have a higher level of education than the general population, and higher educational level is positively related to resilience [48]. However, the resilience of the frontline medical staff who worked in Hubei Province was not significantly higher than that of the general population outside Hubei Province; this may be because the epidemic situation in Hubei Province was more serious, resulting in a decline in resilience.

The current study has several limitations. First, the small number of samples from the general population in Hubei Province limits the ability to draw reliable conclusions regarding the mental health of the public in the region. Hubei Province is the area that has been hardest hit by the epidemic, and the general public there has also been greatly affected [16]. Therefore, more large-scale research on the mental health of the general population in Hubei Province is needed. Second, all the measures relied on self-report questionnaires, which are dependent on individuals’ subjective reports and are vulnerable to reporting bias. Although the PHQ-9, GAD-7, and ISI have been demonstrated to have good diagnostic utility, future research using structured clinical interviews is still warranted. Third, our study had a cross-sectional design. With the development of the epidemic, the psychological symptoms of medical staff and the general population will change. Previous studies have shown that posttraumatic reactions change over time after traumatic events, and individual heterogeneity exists in posttraumatic reactions [49]. Therefore, more longitudinal studies are needed to explore the development of psychological symptoms after the COVID-19 pandemic. Finally, all data were obtained through the WeChat platform. Although the new technology provides the necessary conditions for investigations in situations in which face-to-face contact is not possible, such as during the COVID-19 epidemic and WeChat is widely used in China, individuals who did not know about or were unable to use the WeChat app were not included in the investigation. Therefore, investigations in which data are collected via WeChat app have a bias.

In summary, our findings suggest that frontline medical staff and the general public widely experience symptoms of depression, anxiety, and insomnia. The severity was higher among the medical staff than that in the general public and in people in Hubei Province than in those in other regions. The COVID-19 epidemic is currently spreading around the world [50], and medical staff in many countries are being challenged [43]. It is necessary to provide timely psychological services for the medical staff fighting COVID-19, such as programs to enhance resilience and psychological well-being. For more severely affected areas, more psychological service resources are needed. Meanwhile, the mental health of the general public cannot be ignored. However, the rapid transmission of COVID-19 between people has impeded traditional in-person psychological interventions [51]. Various psychological service channels such as telephone hotlines and online consultations should be developed for the public. During the COVID-19 epidemic in China, online psychological counseling services have been widely established by mental health professionals [51]. Previous research has shown the effectiveness of some online counseling, and the outcome is not different from that obtained through in-person psychological counseling [52]. However, a study of secondary school students in Hong Kong showed that only 45% of the students had an intention to use online counseling services during the COVID-19 epidemic [53]. Therefore, the effectiveness and rate of utilization of these new types of counseling, especially in the context of Chinese culture, should be further explored.

## 5. Conclusions

Frontline medical staff and the general public have widely experienced symptoms of depression, anxiety, and insomnia during the COVID-19 epidemic in China, suggesting that prompt, continuous, and appropriate mental health services are needed both for medical staff and for the general public. The severity of these conditions was higher among the medical staff than among the general public and higher among people in Hubei Province than among those in other regions. Therefore, the allocation of psychological service resources needs to consider susceptible groups and regions in which the epidemic is most severe.

## Figures and Tables

**Table 1:** Characteristics of the participants.

Variable	Medical Workers (*n* = 899)		General Population (*n* = 1104)		*F/*χ^2^
	*N*	Percentage	*N*	Percentage	
**Gender**					36.83 ***
Male	168	18.69%	337	30.53%	
Female	731	81.31%	767	69.47%	
**Age** (years)					198.63 ***
≤20	17	1.89%	215	19.47%	
21–40	607	67.52%	730	66.12%	
41–60	273	30.37%	152	13.77%	
>60	2	0.22%	7	0.63%	
**Marital status**					239.65 ***
Single	214	23.80%	636	57.61%	
Married	636	70.75%	412	37.32%	
Divorced	38	4.23%	41	3.71%	
Other (widowed, separated, etc.)	11	1.22%	15	1.36%	
**Education level**					109.97 ***
Secondary school or below	1	0.11%	20	1.81%	
High school or vocational school	27	3.00%	92	8.33%	
College	112	12.46%	210	19.02%	
University	561	62.40%	676	61.23%	
Master’s	136	15.13%	90	8.15%	
Doctoral degree	62	6.90%	16	1.45%	
**A relative or friend caught the virus** (or was quarantined)	30	3.34%	8	0.72%	18.17 ***
**Annual household income (RMB)**					107.13 ***
30,000–80,000	174	19.35%	449	40.67%	
80,000–300,000	588	65.41%	527	47.74%	
300,000–1,000,000	131	14.57%	117	10.60%	
>1,000,000	6	0.67%	11	1.00%	
**Hours worked per day**					
4–6	79	8.79%			
6–8	362	40.27%			
8–10	373	41.49%			
>10	85	9.45%			
**Years worked**					
≤1	66	7.34%			
2–3	64	7.12%			
4–5	55	6.12%			
6–10	217	24.14%			
11–20	267	29.70%			
>20	230	25.58%			
**Position**					
Infectious disease department	79	8.79%			
Intensive care unit (for quarantine)	58	6.45%			
Emergency department	58	6.45%			
General ward	413	45.94%			
Other (operating room, medical laboratory, etc.)	291	32.37%			

Note: *** *p* < 0.001.

**Table 2:** Mental health of the participants.

Variable	Mean (SD)		Mean (SD)		*F*
	Medical Workers Hubei Province (*n* = 138)	Medical Workers Other Regions (*n* = 761)	General Population Hubei Province (*n* = 30)	General Population Other Regions (*n* = 1074)	
**Depression** **(PHQ-9, range: 0–27)**	7.86 (5.36)	6.33 (5.59)	5.57 (4.71)	5.36 (5.22)	17.10 ***
**Anxiety** **(GAD-7, range: 0–21)**	6.05 (4.61)	4.75 (4.54)	5.03 (5.07)	3.59 (4.25)	28.54 ***
**Insomnia** **(ISI, range: 0–28)**	7.54 (5.88)	5.92 (6.11)	5.80 (5.30)	4.94 (5.55)	15.65 ***
**Resilience** **(CD-RISC-10, range: 0–40)**	26.36 (7.99)	27.47 (8.80)	25.80 (8.10)	26.35 (8.98)	3.76 *

Notes: Because the size of the general population was much smaller in the Hubei Province group than that in the other three groups, this group was excluded when conducting the group differences test. * *p* < 0.05; *** *p* < 0.001. PHQ-9: Patient Health Questionnaire-9; GAD-7: Generalized Anxiety Disorder Scale-7; ISI: Insomnia Severity Index; CD-RISC-10: Connor–Davidson Resilience Scale-10.

**Table 3:** Prevalence of depression, anxiety, and insomnia and their comorbidity.

Variable	Count (percent)		Count (percent)		χ^2^
	Medical Workers Hubei Province (*n* = 138)	Medical Workers Other Regions (*n* = 761)	General Population Hubei Province (*n* = 30)	General Population Other Regions (*n* = 1074)	
**Depression**					
Mild severity	102 (73.91%)	422 (55.45%)	16 (53.33%)	495 (46.09%)	45.10 ***
Moderate severity	42 (30.43%)	176 (23.13%)	7 (23.33%)	196 (18.25%)	14.39 **
**Anxiety**					
Mild severity	83 (60.14%)	341 (44.81%)	16 (53.33%)	346 (32.22%)	57.49 ***
Moderate severity	28 (20.29%)	100 (13.14%)	5 (16.67%)	99 (9.22%)	17.98 ***
**Insomnia**					
Mild severity	60 (43.48%)	226 (29.70%)	7 (23.33%)	252 (23.46%)	28.39 ***
Moderate severity	20 (14.49%)	81 (10.64%)	2 (6.67%)	77 (7.17%)	11.96 **
**Comorbidity**					
Depression + Anxiety	23 (16.67%)	89 (11.70%)	3 (10.00%)	89 (8.29%)	12.46 **
Depression + Insomnia	14 (10.14%)	58 (7.62%)	1 (3.33%)	56 (5.21%)	7.22 *
Anxiety + Insomnia	9 (6.52%)	42 (5.52%)	0 (0%)	33 (3.07%)	8.41 *
Depression + Anxiety + Insomnia	9 (6.52%)	40 (5.26%)	0 (0%)	31 (2.89%)	9.79 **

Notes: Depression + Anxiety: comorbidity of depression and anxiety; Depression + Insomnia: comorbidity of depression and insomnia; Anxiety + Insomnia: comorbidity of anxiety and insomnia; Depression + Anxiety + Insomnia: comorbidity of depression, anxiety, and insomnia. Because the size of the general population was much smaller in the Hubei Province group than that in the other three groups, this group was excluded when conducting the group differences test. * *p* < 0.05; ** *p* < 0.01; *** *p* < 0.001.
